# Gene expression analysis and SNP/InDel discovery to investigate yield heterosis of two rubber tree F1 hybrids

**DOI:** 10.1038/srep24984

**Published:** 2016-04-25

**Authors:** Dejun Li, Rizhong Zeng, Yan Li, Manman Zhao, Jinquan Chao, Yu Li, Kai Wang, Lihuang Zhu, Wei-Min Tian, Chengzhi Liang

**Affiliations:** 1 Rubber Research Institute, Chinese Academy of Tropical Agricultural Sciences, Danzhou 571737, Hainan, China; 2 State Key Laboratory of Plant Genomics and National Plant Gene Research Centre (Beijing), Institute of Genetics and Developmental Biology, Chinese Academy of Sciences, 5 Datun Road, Chaoyang District, 100101 Beijing, China; 3 College of Horticulture & Forestry Sciences, Huazhong Agricultural University, Wuhan, 430070 China

## Abstract

As an important industrial material, natural rubber is mainly harvested from the rubber tree. Rubber tree breeding is inefficient, expensive and time-consuming, whereas marker-assisted selection is a feasible method for early selection of high-yield hybrids. We thus sequenced and analyzed the transcriptomes of two parent rubber trees (RRIM 600 and PR 107) and their most productive hybrids (RY 7-33-97 and RY 7-20-59) to understand their gene expression patterns and genetic variations including single nucleotide polymorphisms (SNPs) and small insertions/deletions (InDels). We discovered >31,000 genetic variations in 112,702 assembled unigenes. Our results showed that the higher yield in F_1_ hybrids was positively associated with their higher genome heterozygosity, which was further confirmed by genotyping 10 SNPs in 20 other varieties. We also showed that RY 7-33-97 and RY 7-20-59 were genetically closer to RRIM 600 and PR 107, respectively, in agreement with both their phenotypic similarities and gene expression profiles. After identifying ethylene- and jasmonic acid–responsive genes at the transcription level, we compared and analyzed the genetic variations underlying rubber biosynthesis and the jasmonic acid and ethylene pathways in detail. Our results suggest that genome-wide genetic variations play a substantive role in maintaining rubber tree heterosis.

Natural rubber (NR) is the critical raw material for more than 40,000 products, including 400 medical devices^1^. It cannot be replaced by synthetic alternatives due to its unique properties, such as resilience, elasticity, impact and abrasion resistance, efficient heat dispersion and malleability at cold temperature^2^. While *Hevea brasiliensis* (the para rubber tree), of the Euphorbiaceae family, is perennial and native to Amazon rainforests, rubber trees are now mainly cropped in South and Southeast Asia. Similar to other Euphorbiaceae species, the rubber tree produces lateral inflorescences, and is a monoecious, self- and cross-pollinated^3^,^4^ and diploid species (2n = 36, n = 18) with a C value of ~2 pg (2.15 Gb of haploid genome)^5^. *H. brasiliensis* (the para rubber tree) is the only species cultivated commercially and is the primary source of NR. With the rapid development of the world economy, the global demand for NR will be increasing till 2020 and possibly beyond (http://www.dailymirror.lk/business/features/31402-meeting-increasing-global-demand-for-natural-rubber.html). Breeding new varieties and developing new tapping techniques are the most effective approaches to optimize latex regeneration and increase NR production from rubber trees. However, the yield genetic improvement of rubber tree is very inefficient and time-consuming mainly due to the difficulty to dissect yield component traits. Although great progress has been made in yield breeding of rubber tree since the 1920s, the yield of the most productive varieties currently available is still much inferior to the theoretical yield of rubber tree, which was predicted to be 7,000–12,000 kg/ha/yr^6^.

It is widely accepted that the three key factors that determine rubber yield^7^ are the number of secondary laticifers, latex regeneration between successive tappings, and the duration of latex flow. It has been proposed that the mevalonate and 2-C-methyl-d-erythritol 4-phosphate pathways are responsible for providing isopentenyl diphosphate for rubber biosynthesis^8^. Our previous studies have demonstrated that jasmonate (JA) hormones are involved in regulating the secondary laticifer differentiation and latex production^9^,^10^,^11^,^12^. These findings are consistent with the crucial role of JAs in regulating secondary metabolism^13^. Several genes have been suggested as limiting factors in rubber biosynthesis^14^,^15^,^16^,^17^. In addition, latex coagulation is associated with different kinds of proteins and is a decisive factor that causes cessation of latex flow^18^,^19^,^20^, whereas the application of Ethrel, a releaser of the ethylene (ET), is very effective in prolonging the duration of latex flow^21^; therefore, the genes involved in latex coagulation and ET signaling are also associated with rubber yield.

The combination of conventional and modern breeding technologies is proving useful for improving the efficiency of rubber tree breeding^22^. As an important component of modern breeding technologies, molecular markers have been developed and widely used in the field in such techniques as marker-assisted selection (MAS), DNA fingerprinting, population genetics, genetic diversity, gene flow, and genetic mapping, etc. However, the fact that genomic and transcriptomic resources are limited has effectively restricted the application of MAS in rubber tree breeding. The next-generation sequencing method provides a powerful means of generating large sequence datasets that can be used to characterize sequence diversity^23^ and to generate the necessary polymorphic and genotypic data for genetic mapping^24^, genetic diversity analysis, gene identification, and molecular breeding^25^,^26^. Single nucleotide polymorphisms (SNPs) and small insertions and deletions (InDels) identified in next-generation sequencing data have been widely used in genome and transcriptome analyses of human and many other animal species and in plants. For the rubber tree, the available transcriptome data have been generated from latex and leaves, bark, and shoot apical meristem with next-generation sequencing methods^27^,^28^,^29^. Although a draft genome of the rubber tree has been completed by Malaysian researchers^30^, the fine genome sequence is still not available as a reference for many possible omics and genetic studies and MAS applications to the rubber tree.

New rubber tree varieties have been bred mainly through traditional cross-breeding programs in rubber-producing countries. The rubber tree clones PR 107 (a primary variety) and RRIM 600 (a secondary variety) are usually used as the backbone parents in cross-breeding owing to their favorable features. RRIM 600 was selected from the offspring of the primary varieties Tjir 1 and PB 86^31^. To date, many rubber tree varieties with high-yield potential have been selected from hybrids of RRIM 600 and PR107. In China, two elite clones, RY 7-33-97 and RY 7-20-59, were bred using RRIM 600 and PR107 as the female and male parents, respectively, through artificial pollination and selection^32^. Studies have demonstrated that the hybrids RY 7-33-97 and RY 7-20-59 are superior to their parents in a number of agricultural phenotypes, especially in latex yield. The underlying genetic basis for this finding needs to be elucidated for further MAS application in rubber tree breeding.

In this study, we used RNA-seq to investigate the genomes of the parents (RRIM 600 and PR 107) and their two elite hybrid offspring RY 7-33-97 and RY 7-20-59. Using assembled unigenes as reference, we identified the SNPs and small InDels in genes that are involved in latex metabolism and the JA and ET pathways. Furthermore, we randomly selected 33 genes for detailed analysis of their expression profiles in the two hybrids and their parents. Based on these data, the genetic makeup and variations between the parents and their elite hybrid progeny were compared and analyzed with respect to their transcriptomes and three major pathways related to rubber yield.

## Results

### Illumina Sequencing, *De Novo* Assembly, and Functional Classifications

**Figure 1:**
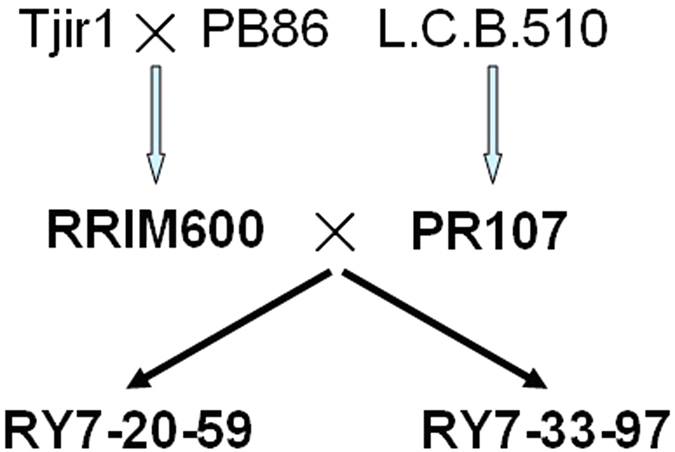
Genetic background of the four rubber tree varieties. The female parent of each cross is listed first. The four rubber tree varieties sequenced in this study are in bold type.

The relationship of the four rubber tree varieties (PR 107, RRIM 600, RY 7-33-97 and RY 7-20-59) is illustrated in Fig. 1, and the differences they show in three traits related to latex yield are listed in Table 1. RNA-seq data were obtained from samples of latex from PR 107, RRIM 600, RY 7-20-59, and RY 7-33-97 and from a mixture of latex and leaves from RY 7-33-97. All raw sequence data were deposited in NCBI Biosample with the accession numbers SAMN00254193 and SAMN03568829–03568838. After assembly, 112,702 unigenes were obtained, with an average length and N50 of 712 bp and 1,248 bp, respectively. Of these unigenes, 60,389 showed amino acid sequence identity to proteins in the NR, Swiss-Prot, KEGG, and COG databases with an e-value cutoff of 1e-5 (Table 2). Coding sequence completeness of the assembled unigenes was defined by the presence of start and stop codons. In total, 20,273 unigenes (33.6%) contained both and therefore were designated as full unigenes, whereas 23,318 (38.6%) had only one or the other and were thus classified as partial (Table 2). Using homologous proteins as standards, we further characterized the putative unigenes and found 2,359 (3.9%) as misassembled (multiple genes assembled to one unigene) and 2,811 (4.7%) as split (two partial unigenes belonging to the same gene), which suggested that the error rate in the assembly was minimal. Finally, we mapped the unigenes to the draft genome^30^ and found that 98,278 (84.6%) mapped with >50% coverage (BLAT identity cutoff 95%). The unigenes were assigned to different functional categories with Blast2GO, and the annotations were manually verified and integrated by gene ontology (GO) classification.

### Identification of ET- or JA-responsive Genes in Rubber Tree

**Figure 2:**
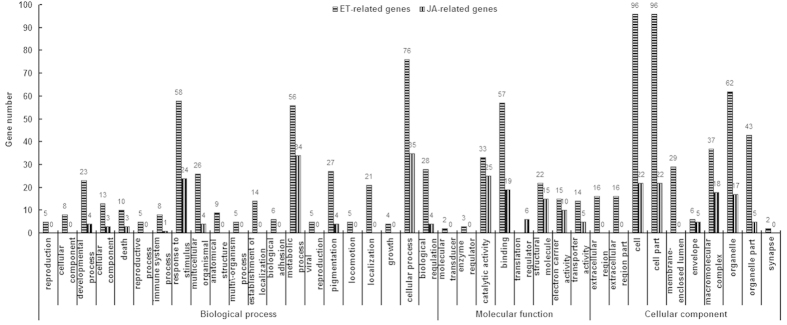
Enriched GO pathways of ET and JA-related genes.

ET and JA are very important hormones for the rubber tree. Although Ethrel has been extensively used as a powerful stimulant of latex yield for over half a century, the molecular mechanisms by which ET promotes latex flow and increases latex production, and how the different rubber tree varieties each respond to Ethrel stimulation, remain elusive. The situation is even worse in the case of JA where far less is known about the physiological and molecular aspects of its function in the rubber tree. Using the assembled unigenes as reference, we analyzed clean reads from the latex of rubber trees treated with ET or JA, and the appropriate controls, for differentially expressed genes (DEGs). A total of 679 DEGs were identified as ET responsive, and of these 488 and 191 were up- or downregulated, respectively; 310 DEGs were JA responsive, and of these 173 and 137 were up- or downregulated, respectively. Among the ET- and JA-responsive DEGs, 198 were involved in both the ET and JA responses, suggesting that these DEGs are co-regulated by ET and JA in the rubber tree (Supplementary Dataset 1). As shown in Fig. 2, the ET-related DEGs were significantly enriched in 38 GO terms, such as response to stimulus, cell, binding. The JA-related DEGs were significantly enriched in 22 GO terms including response to stimulus, metabolic process, cell, cell part, for instance. By comparing these DEGs with those involved in the ET and JA pathways from *Arabidopsis*, we identified 73 and 29 genes, respectively, that putatively function in the ET and JA pathways in the rubber tree. Of the 29 genes involved in the JA pathway, 19 also are involved in the ET pathway (Supplementary Dataset 2).

### Landscape of Genetic Variations among the Four Varieties

**Figure 3:**
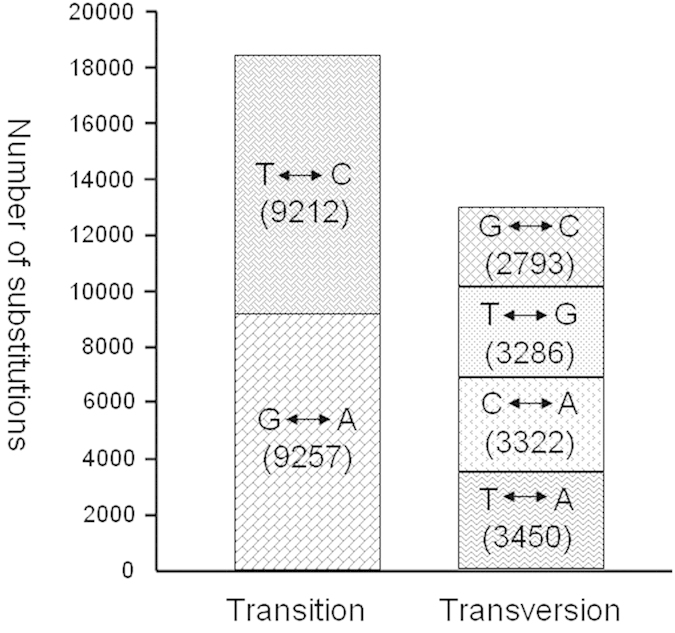
Classes of SNP detected between RRIM 600 and PR 107. The number of each type of single-base substitution is indicated in parentheses.

The genetic variations (SNPs and small InDels) between PR 107 and RRIM 600 were individually identified by Samtools and the Genome Analysis Toolkit GATK (see Methods). To increase prediction accuracy, only the variations predicted by both methods were used for subsequent analyses. We detected 31,320 SNPs in 9,764 unigenes in total (Supplementary Dataset 3). As shown in Fig. 3, >58.97% (18,469) of nucleotide variants were transitions, and the remaining 41.03% (12,851), transversions. The transitions between G and A were equal to those between T and C. For the transversions, the substitutions arranged in order of decreasing frequency were between T and A, C and A, T and G, and G and C. According to their position in unigenes, SNPs are divided into four types: coding region, 5′ untranslated region, 3′ untranslated region, and unknown position (when no coding sequence in a unigene was identified); we identified 19,569, 2,397, 5,069 and 4,285 SNPs for each category, respectively (Table 3). The SNPs assigned to the coding region were classified as synonymous or non-synonymous, with a corresponding ratio of ~0.89, suggesting that a large number of the DEGs may have functional differences between the two parents. Only 140 unigenes contained small InDels (Supplementary Dataset 3), of which 17 were located in coding regions (Table 3).

We further analyzed the genetic variations shared by all four elite rubber tree varieties. Among 9,043 unigenes expressed in all four, 7,975 (88.19%) contained 24,875 SNPs (Supplementary Dataset 4). We randomly selected 408 SNPs (from 102 loci) to validate the prediction accuracy and found that 377 (92.4%) were consistent with the predicted results, suggesting that the results of SNP analyses were highly accurate (Supplementary Dataset 5). To evaluate the influence of alternative splicing on SNP calling, we further compared the same 408 SNPs examined by Sanger sequencing with the SNPs called using the genomic sequence of RRIM 600 as a reference. The SNPs were divided into two categories: 87 loci (348 SNPs) were mapped to the genomic sequence of RRIM 600 (at least 70% of their reference unigenes in length was mapped to the genomic sequence), and the other 15 loci (60 SNPs) were not (due to the incompleteness or fragmented nature of the genomic sequence). While the SNP calling accuracy for the former category was 91.67%, it was 96.67% for the latter. Among the 87 loci mapped to the genomic sequence, 79 loci were also identified by using the genomic sequence as a reference, and the prediction accuracy on these 79 loci was 90.82%. These results suggested that the SNP calling reliability was relatively high with the transcriptome assembled in this study as the reference sequence. Among the 24,875 SNPs, the heterozygous percentages of RY 7-33-97, RY 7-20-59, PR 107 and RRIM 600 were ~70.52%, 63.13%, 51.97%, and 53.60%, respectively (Table 4); thus, there was a much higher heterozygosity ratio in the offspring varieties. We found only 113 small InDels among the 9,043 unigenes (Supplementary Dataset 4). The accuracy of small InDel prediction was ~80.00% (Supplementary Dataset 5). The heterozygous percentages regarding the InDel sites of RY 7-33-97, RY 7-20-59, PR 107 and RRIM 600 were ~7.96%, 7.96%, 14.16%, and 20.35%, respectively (Table 4).

We next inspected the hybrid and parent sequences to determine the frequency of shared genetic variations. When compared, respectively, to the parental transcriptomes of RRIM 600 and PR 107, the hybrid RY 7-33-97 shared 9,128 and 7,366 SNP genotypes, and 30 and 38 small InDels; in the same comparison, the hybrid RY 7-20-59 shared 7,868 and 8,626 SNP genotypes and 38 and 30 small InDels (Table 5). Among these genetic variations, a subset was shared with both parents: for RY 7-33-97 these included 3,393 SNPs and 21 small InDels, and for RY 7-20-59, 3,847 SNPs and 21 small InDels. There were also a number of genetic variations that were specific to the hybrids; for RY 7-20-59 these included 4,988 SNP genotypes and 24 small InDels, and for RY 7-20-59, 5,534 SNP genotypes and 24 small InDels (Table 5). Based on the genetic variations shared by each offspring hybrid and its parents, RY 7-33-97 and RY 7-20-59 were closer in genetic content to RRIM 600 and PR 107, respectively. This may partially explain why many observed phenotypes of 7-33-97 were closer to RRIM 600 and those of 7-20-59 to PR 107, including ET and JA responses (Table 1).

### Genome Heterozygosity May Positively Contribute to Latex Yield

Taken together, the results in Tables 1 and 4 clearly indicate that the rubber yield is positively correlated with the SNP heterozygosity ratio in the four varieties, which suggests that heterosis plays an important role in increasing rubber yield. This may not be significant, however, owing to the small number of varieties we tested. To explore this further, we selected 10 SNPs within four genes involved in latex biosynthesis and the related flow pathway for experimental validation in the four varieties, and we included 20 other high-yielding rubber tree varieties that themselves are secondary or tertiary varieties selected from the progenies after two or three hybridizations. As shown in Table 6, we found that the polymorphism information content for each SNP ranged from 0.2188 to 0.5729, with the average being 0.3824. The overall SNP heterozygosity percentages of the 24 rubber tree varieties varied from 0 to 100% with an average of 68.75%. The heterozygosity percentage of all three primary varieties (Tjir 1, PR 107 and PB 86) was <50%; in contrast, the heterozygosity percentage of 17 of 21 (>80%) secondary or tertiary varieties was >60% (Table 6). Although we do not know the exact yields of the other 20 varieties, we hypothesize that rubber tree varieties were selected mainly based on their latex yields. In any case, the high yield in the selected offspring hybrids is positively correlated with genome heterozygosity, suggesting that high genome heterozygosity may play an important role in increasing latex yield in rubber tree breeding.

### Genetic Variations between Elite Hybrids and Their Parents Concerning Three Pathways

The pathways associated with ET, JA, and latex biosynthesis and flow (LBF) are strongly linked to rubber tree yield. Therefore, we further analyzed the genetic variations between the hybrids and their parents in genes that are involved in these three pathways. First, among 34 latex biosynthesis- and flow-related genes, 18 were found to contain 33 SNPs (see Table 7 and Supplementary Dataset 6 for details). This is very similar to the results of Mantello *et al.*^33^, except for the hydroxymethylglutaryl-CoA synthase gene which did not contain SNPs in this study. Second, 47 SNPs were identified in 20 genes involved in the ET pathway (Table 7 and Supplementary Dataset 6). Finally, we found 18 SNP sites located in 8 JA pathway genes (Table 7 and Supplementary Dataset 6). In general, as expected in the F_1_ hybrids, there were fewer SNPs that were either the same as, or different from, both of their parents, than the number that was shared with only one parent.

### Expression Profiles of 33 Randomly Selected Genes Compared between Elite Hybrids and Their Parents

**Figure 4:**
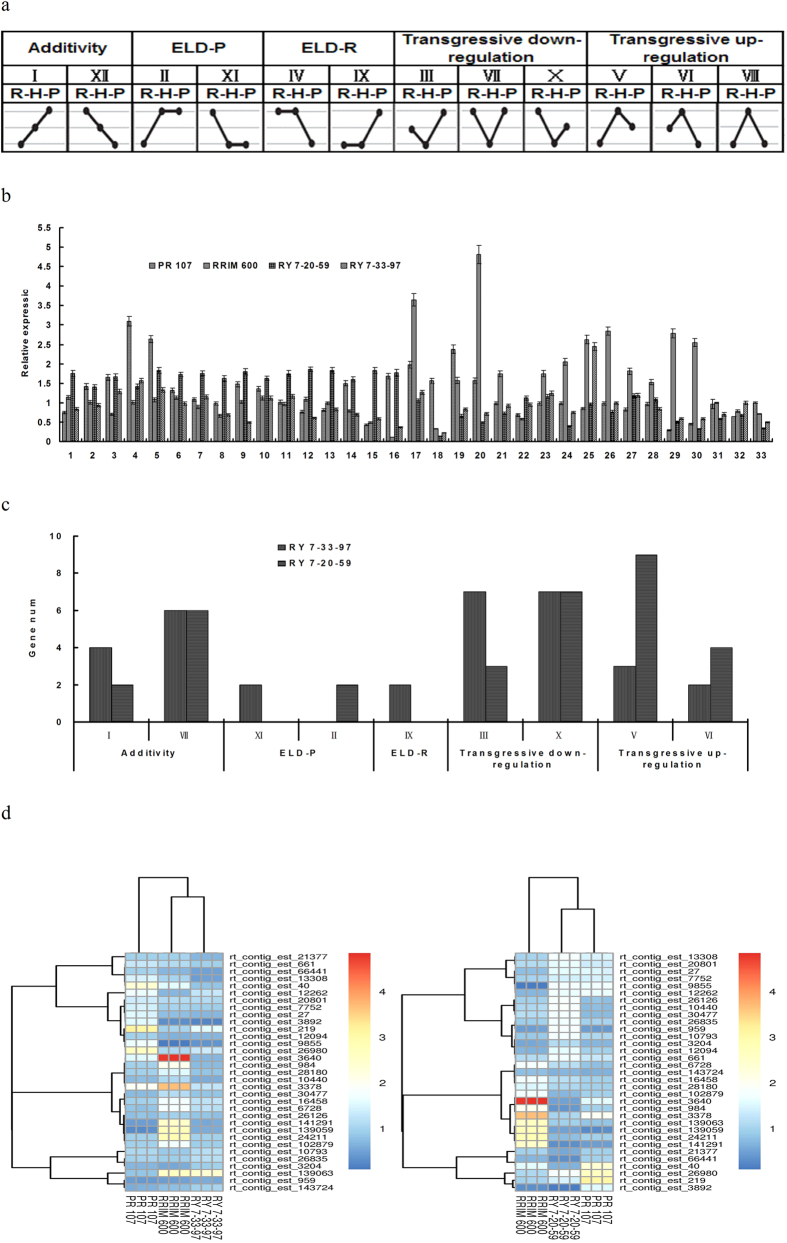
Expression analyses of 33 genes between elite hybrids and their parents. (**a**) Twelve bins of DEGs. R, P, and H represent RRIM 600, PR 107, and the hybrids, respectively. (**b**) Expression patterns of 33 genes between the elite hybrids and their parents. (**c**) Number of genes showing different types of expression corresponding to the bins in (**a**) in RY 7-33-97 and RY 7-20-59. (**d**) Images of 33 genes bidirectionally clustered by pheatmap software according to differential gene expression between the hybrids and their parents.

The impact of intraspecific allelic variations on gene expression may lead to phenotypic variation, including the possibility of hybrid vigor as a beneficial trait that is exploited in crop breeding^34^,^35^. Because the combined allelic expression may deviate from that of either parent or the mid-parent prediction in a hybrid^36^, we randomly selected 33 latex-expressed genes with or without genetic variations to analyze their expression levels in the hybrids and their parents. Based on the expression patterns shown in Fig. 4a, these genes were further classified into 12 groups according to their differential expression in the hybrids and parents (R-H-P_dif_; Fig. 4b). Additionally, we classified the hybrid genes with respect to their expression level dominance (ELD): those with expression statistically similar to that in RRIM 600 were defined as ELD-R genes and those similar to PR 107 as ELD-P genes. In RY 7-33-97, ~30% (10/33) of genes were additive, and the remaining 70%, (23/33) were non-additive. Of the latter, ~42% (14/33) showed a transgressive downregulation pattern, followed by ~21% with transgressive upregulation (5/33); with respect to ELD, ~6% (2/33) were ELD-P, and ~6% (2/33) were ELD-R (Fig. 4c). As for RY 7-20-59, the genes exhibiting additivity and non-additivity represented ~24% and 76% of the total, respectively. The latter genes were divided into three groups: ELD-P, transgressive upregulation, and transgressive downregulation, for which the percentages were, ~6% (2/33), 39% (13/33), and 30% (10/33), respectively (Fig. 4c). In addition, the 33 genes were bidirectionally clustered according to the differential gene expression observed between the hybrids and their parents. As shown in Fig. 4d, the expression patterns of 33 genes were classified into three clusters for RY 7-20-59 and RY 7-33-97. The expression profiles of genes in RY 7-33-97 were more similar to those in RRIM 600, whereas those in RY 7-20-59 were more similar to those in PR 107. Thus, the clustering was consistent with the genetic variations previously detected between the hybrids and their parents.

## Discussion

All the rubber tree cultivars that have been extensively planted around the world were bred and selected from the Wickham germplasms, a sample population substantially modified by human selection and bred for only approximately one century^37^. Numerous rubber tree varieties with higher latex yield have been selected from the hybrids of several backbone parents, but the successive breeding programs may have caused decreased genetic variation in the Wickham germplasms and thus led to a reduced potential for further latex production increases. Herein, we present a pedigree analysis of the genetic variations and gene expression patterns of four elite varieties, which provides much more information regarding the underlying genetic compositions and molecular mechanisms related to the latex production of rubber trees than that furnished by recently published reports using only one or two varieties of *H. brasiliensis*^33^,^38^. We found that the transcriptomes of offspring varieties RY 7-33-97 and RY 7-20-59 were more similar to those of the parental varieties RRIM 600 and PR 107, respectively, both in SNPs and gene expression levels. This is consistent with the observation that the phenotypes of RY 7-33-97 and RY 7-20-59 were also more similar to RRIM 600 and PR 107, respectively^39^,^40^. Furthermore, compared with their parents, there were a large number of SNP genotypes that proved specific to either RY 7-33-97 or RY 7-20-59. All the specific SNP genotypes (heterozygous or homozygous SNPs) in RY 7-33-97 or RY 7-20-59 come from the independent assortment of different homozygous gametes and same heterozygous gametes from their parents. These new SNP genotypes may lead to more productive phenotypes in the hybrids than in their parents. Indeed, RY 7-33-97 and RY 7-20-59 showed many physiological differences from their progenitors, such as the number of volatile fatty acids, molecular weight/10^4^, stretching length ratio, etc.^39^,^40^. Our results suggest that the Wickham germplasms are highly heterozygous, which might somewhat offset the shortage of genetic resources that seem to have been generated by rubber tree breeding. Therefore, we conclude that the Wickham germplasms may continue to be used in breeding with elite rubber tree varieties as hybrid parents through artificial pollination and selection procedures.

In this study, more than 31,000 putative SNPs in genic regions were detected among the four elite varieties. By analyzing these SNPs, we found that the genetic heterozygosity of the latex transcriptome of the hybrids was significantly greater than that of their parents and correlated with the latex yield of different rubber tree varieties. This implies that the SNP variations in the filial generations may play a significant role in the manifestation of heterosis^41^,^42^, which contributes to increased latex yield of the F_1_ hybrids. Our experimental validation of 10 SNPs in 24 rubber tree varieties, including 21 secondary or tertiary varieties and 3 primary varieties, showed that the heterozygous percentages of secondary or tertiary varieties are generally greater than those present in primary varieties. This suggests that rubber tree varieties selected by breeders tend to have greater genome heterozygosity or that hybrid vigor has been extensively exploited in rubber tree breeding.

Intraspecific hybridization between two cultivars or accessions can result in up- or downregulation of gene expression^43^. Compared with the same genes in their parents, 33 randomly selected genes exhibited both additive and non-additive expression patterns in RY 7-33-97 and RY 7-20-59. Several studies reported a high proportion of non-additive expression patterns in F_1_ hybrids^43^, which is consistent with our results that a majority of the DEGs exhibited non-additive gene action in RY 7-33-97 (70%) and RY 7-20-59 (74%). Among those genes that were non-additively expressed, the proportion of the DEGs with overdominant action was greater than that found in comparable data from prior reports^41^,^44^. Although heterosis may be controlled by many genes, only a small fraction of those genes are actually involved^36^. It is possible that only some of the DEGs between F_1_ hybrids and their parents may contribute to the superiority or heterosis of rubber tree hybrids as has been reported for rice, maize, and pufferfish^43^,^45^,^46^. We also found that some DEGs exhibited dominant and additive actions. Several researchers have suggested that heterosis is attributable to the orchestrated outcome of partial-to-complete dominance, overdominance, and epistasis^43^,^45^,^46^,^47^,^48^. As a complex quantitative trait, latex yield is likely to be under the influence of multiple factors; in addition to overdominance, other possibilities include epistasis, gene-environment interactions, maternal effects. The results from our present study form part of a new framework with which to understand and explore the contributions of heterosis to latex yield and highlight specific areas requiring further research.

Four main categories of genetic changes may be considered to modify gene products: 1) premature stop codons; 2) alterations of the initiating methionine residue; 3) induction of frame-shift mutations; and 4) removal of annotated stop codons. We found that a majority (367) of the large-effect SNPs likely altered the initiating methionine residues. Because large-effect genetic variations in genic regions operate as deleterious variations, they have potentially disabling effects on gene function or the integrity of encoded proteins; as such, they have been suggested as a genetic basis contributing to heterosis^49^,^50^. Although premature stops are rare and often detrimental, these loss-of-function variations are particularly likely to underlie many of the traits selected during domestication of numerous crop plants^49^.

Owing to the important roles of three pathways, namely ET, JA, and latex production/flow, in rubber tree yield, we targeted the genetic variations underlying these pathways between the hybrids and their parents as likely major causal factors influencing output. We identified 33 SNPs in 18 genes involved in LBF pathways. Mantello *et al.*^33^ also analyzed SNP markers for the rubber biosynthesis pathway. Compared with other plants, factors affecting the binding of ET-responsive elements were overrepresented in the rubber tree^30^,^51^, and SNP variations were indeed detected in the genes underlying the ET pathway in our study. Therefore, the SNPs in the genes involved in the three pathways may be correlated with the phenotypic differentiation in four rubber tree varieties, particularly because the SNPs are inherited genomic point mutations, and SNP variants can greatly influence transcript expression levels^52^,^53^.

JA and its conjugate, methyl jasmonic acid, contribute to diverse biological functions in plants^54^, and each acts as a conserved elicitor of secondary metabolism in a wide range of plant species^13^. Although the underlying mechanism by which JA promotes rubber production has been studied less than that of ET, based on the laticifer differentiation and rubber biosynthesis–related genes regulated by JA^9^,^12^, we postulate that JA may be a pivotal regulator of rubber biosynthesis specifically because it is a typical isoprenoid secondary metabolite that utilizes isopentenyl pyrophosphate as a biosynthetic precursor^55^. In our study, we identified many genes underlying ET and JA pathways in rubber tree. This is the first time in the rubber tree that the ET- and JA-related genes were identified at the latex transcriptome level, which will enable us to further understand the roles of ET and JA in rubber tree latex synthesis.

Plant hybridization is a common process in nature and plays a vital role in plant breeding. As a perennial tree with a long growing and breeding cycle, conventional breeding of the rubber tree is labor-intensive and time-consuming. With heterozygous parents, it was expected, and in fact shown, that the hybrid progenies in F_1_ populations are still rich in genetic variations. Therefore, it is necessary for rubber tree breeders to use an effective selection method (e.g., MAS) to increase breeding efficiency as opposed to the traditional pure phenotype-based selection process. The SNP variations in genic regions (especially in genes in critical pathways) may play an important role in the manifestation of heterosis, which in turn may contribute to increased latex yield in F_1_ rubber tree hybrids. Therefore, our transcriptome-wide survey of SNPs and small InDels in four elite rubber tree varieties provides potential new strategies for the genetic-based improvement of rubber tree yield during breeding, i.e., increasing latex yield by selecting highly heterozygous offspring in the seedling stage from hybrids of elite parents via MAS. Our future work shall focus on the relationship between the SNPs/small InDels and corresponding gene expression changes that may lead to phenotypic variations. To further explore the relationship between genome heterozygosity and rubber yield, we will construct an F_1_ population using the four heterozygous elite varieties. The SNPs and small InDels discovered in this study will facilitate the construction of high-density genetic maps based on the F_1_ population, as shown by Pootakham *et al.*^56^ and Shearman *et al.*^57^, which can be further used for MAS breeding or other methods such as quantitative trait locus mapping.

## Methods

### Plant Materials, RNA and DNA Extraction

Latex was collected from four rubber tree varieties at the Guangba experimental farm in Hainan, China and dropped directly into liquid nitrogen for total RNA extraction and subsequent Illumina sequencing^58^. RNA quality was assayed with a 2100 Bioanalyzer (Agilent Technologies). Young leaves of 20 other rubber tree varieties were collected from the National Rubber Tree Germplasm Repository, China. Leaf tissue (2 g) was ground in liquid nitrogen and genomic DNA extracted using the CTAB method.

### Illumina Sequencing, *De Novo* Transcriptome Assembly and Annotation

Sequencing library construction and Illumina sequencing were performed at Beijing Genomics Institute–Shenzhen, China. Low-quality reads were filtered out prior to assembly based on the following standards: (1) percentage of low-quality bases (quality value ≤ 5) was >50% in a read; (2) the percentage of N bases was >10% in a read; (3) reads with the adaptor sequence; (4) reads with unmatched paired-ends after the above three steps.

The high-quality reads from RY 7-33-97 were assembled according to the Trinity method. The assembled sequences were further incorporated into distinct contigs using the programs Cap3 and cdhit^59^,^60^. All contigs were subjected to BLAST analysis against the NCBI nonredundant database to remove contaminating sequences and further trim vector contamination. During the *de novo* transcriptome assembly, the Trinity method used default parameters: the parameter in cap3 was -p 98 -o 50, and the parameter in cdhit was -c 0.98 -G 0 -aS 0.90 -AS 200 -M 0 -g 1.

The assembled unigenes were functionally annotated by BLAST against NCBI nonredundant, Swiss-Prot, KEGG, and KOG databases with an e-value cut-off of 10^–5^. When the annotation and open reading frame prediction for a unigene were in conflict among these four databases, the priority was given in the following order: NCBI nonredundant, Swiss-Prot, KEGG and KOG. All the assembled contigs were aligned with the NCBI nonredundant database with the sBLASTX program to predict the position of SNPs and small InDels in a target gene.

### Identifying ET- and JA-related Genes in Rubber Tree

With the assembled unigenes as a reference transcriptome, the DEGs between the controls (no treatments) and ET- or JA-treated materials were identified with TopHat and Cuffdiff 61,62. The criterion for selecting DEGs was a *q*-value cut-off of 0.05, and the absolute log_2_ (fold change) value was >1 after normalization. The ET- and JA-related genes were defined as the DEGs between the ET- and JA-treated materials and the controls, respectively. The enriched KEGG and GO pathways of the DEGs were separately determined using kobas software^63^ and Blast2GO (http://www.blast2go.org/), respectively. In this study, the KEGG and GO pathways showing a corrected p-value ≤ 0.05 were considered to be significantly enriched. To further analyze the genes involved in the ET and JA pathways, the sequences of genes that function in these pathways in *Arabidopsis* were downloaded (http://ahd.cbi.pku.edu.cn/) and aligned to the sequences of the DEGs from rubber tree with e-value < E-10. The aligned genes were considered as those underlying ET and JA pathway function in the rubber tree.

### Discovery, Analysis and Validation of SNP and Small InDels

With the assembled transcriptome as a reference, the latex data from PR 107, RRIM 600, RY 7-33-97, and RY 7-20-59 were aligned for SNP and small InDel identification. Two reliable and frequently used software programs, Samtools and GATK, were independently applied for the identification of SNPs and small InDels with the assembled contigs as a reference sequence. As for small InDels, the number of insertions and deletions ranged from 1 to 6 bp. The filtering thresholds were set as following: the consensus quality was no less than 50 and combined-sample read depth was no less than 5. The SNPs and small InDels identified in both Samtools and GATK outputs were manually corrected according to the genotype relationship between the hybrids and their parents based on Mendel’s laws, and only those that were identified in all the four varieties were regarded as the SNPs and small InDels. When the genotypes of genetic variations in the hybrids were abnormal, the corrected SNPs and small InDels were regarded as reliable genetic variations for the subsequent analyses. The degree of SNP polymorphism of 24 rubber tree varieties was calculated using polymorphism information content^64^.

To validate the accuracy of SNP prediction, the unigenes containing SNPs were selected to design PCR primers and amplified with the designed primers. The PCR products were sequenced from both directions and the chromatograph flies were analyzed by the Chromas software; if the sequencing results of SNP sites contain one and two nucleotides with high-quality, the SNP sites are defined as homozygous and heterozygous ones, respectively. The sequencing results from four rubber tree varieties were aligned and validated the accuracy of predicted SNPs. In addition, 10 SNPs within 4 genes involved in LBF pathway were amplified, sequenced and analyzed in 24 rubber tree varieties. To validate the accuracy of predicted small InDels, the unigenes containing small InDels were selected to design CAPS or dCAPS primers and amplified. The PCR products were digested with their corresponding enzymes, and the digested products were detected by agrose gel electrophoresis. The primers designed for validating small InDels and SNPs were shown in Supplementary Dataset 5 and 7.

The genetic variations (SNPs and small InDels) within the genes involved in *Arabidopsis* ET- and JA pathways as well as LBF were identified by aligning their sequences with the assembled unigenes containing genetic variations with e-value < 1.0E-95. The genes underlying ET, JA pathway were shown in Supplementary Dataset 2. The names and accession numbers of thirty-four genes involved in LBF were shown in Supplementary Dataset 8. As the synonymous SNPs from coding region did not change the sequences of amino acids, they were excluded in subsequent analyses.

### Real-time reverse transcription (RT)-PCR Analyses

For each real-time RT-PCR reaction, Supplementary Dataset 9 lists the gene-specific primers and the primers of the 33 internal reference genes that were used for expression analyses. The PCR cycling program was as follows: 94 °C for 30 s for denaturation, followed by 45 cycles at 94 °C for 5 s, 60 °C for 20 s, and 72 °C for 20 s. The relative abundance of transcripts was calculated with LightCycler Relative Quantification Software 4.05. All real-time RT-PCR experiments described here were reproduced >3 times using independent cDNA preparations, and the values are presented as mean ± S.D.

The gene expression profiles were classified into 12 groups according to their differential expression patterns among the hybrids and their parents (R-H-P_dif_)^65^. We designated the hybrid genes as a function of their ELD in H (hybrids): hybrid genes statistically similar to those in RRIM 600 were termed ELD-R genes, and those similar to PR 107 as ELD-P genes. The 33 genes were bidirectionally clustered according to their expression profiles between the hybrids and their parents by pheatmap software (R package).

## Additional Information

**Accession codes:** The accession numbers of all the raw sequence data in the NCBI Biosample are SAMN00254193 and SAMN03568829-03568838, and the accession number of TSA is GDFU00000000.

**How to cite this article**: Li, D. *et al.* Gene expression analysis and SNP/InDel discovery to investigate yield heterosis of two rubber tree F1 hybrids. *Sci. Rep.* **6**, 24984; doi: 10.1038/srep24984 (2016).

## Supplementary Material

Supplementary Information

Supplementary Dataset 1

Supplementary Dataset 2

Supplementary Dataset 3

Supplementary Dataset 4

Supplementary Dataset 5

Supplementary Dataset 6

Supplementary Dataset 7

Supplementary Dataset 8

Supplementary Dataset 9

## Figures and Tables

**Table 1:** The latex yield-related traits of four rubber tree varieties.

RTV	PR 107	RRIM 600	RY 7-20-59	RY 7-33-97
DRP (kg/hm^2^)	965.5	1257	1467	1983
DRC (%)	33.5	29.8	31.2	33.7
RES	insensitive	sensitive	similar to PR 107	similar to RRIM 600

RTV, DRP, DRC and RES represent rubber tree varieties, dry rubber production, dry rubber content and response to ET stimulation, respectively.

**Table 2:** Transcriptome assembly and annotation statistics of assembled unigene.

Unigene number	112,702
Total length (bp)	80,347,241
Max length (bp)	15,863
Min length (bp)	201
Average length (bp)	712
N50 (bp)	1,248
N90 (bp)	280
Number of annotated unigenes	60,389
Unigenes with complete ORF	20,273
Unigenes with only start or stop codon	23,318
Split unigenes	2,359
Misassembled unigenes	2,811

**Table 3:** Positions of genetic variations predicted in rubber tree unigenes.

Positions	Number	
	SNP	small InDel
5′ UTR	2,397	36
3′ UTR	5,069	64
coding region	19,569	17
unknown	4,285	23

**Table 4:** Genetic makeup of the four rubber tree varieties.

G	SNP								Small InDel							
	PR 107		RRIM 600		RY 7-20-59		RY 7-33-97		PR 107		RRIM 600		RY 7-20-59		RY 7-33-97	
	N	P	N	P	N	P	N	P	N	P	N	P	N	P	N	P
0/0	6,974	28.04%	8,949	35.97%	7,174	28.84%	6,138	24.68%	37	32.74%	30	26.55%	22	19.47%	24	21.24%
1/1	4,974	20.00%	2,593	10.42%	1,998	8.03%	1,196	4.81%	60	53.10%	60	53.10%	82	72.57%	80	70.80%
0/1	12,927	51.97%	13,333	53.60%	1,5703	63.13%	1,7541	70.52%	16	14.16%	23	20.35%	9	7.96%	9	7.96%

G, N and P represent genotypes, number and percentage, respectively.

**Table 5:** The characteristics of genetic variations between elite hybrids and their parents.

The characteristics of genetic variations	Rubber tree hybrids	
	RY 7-33-97	RY 7-20-59
Concordant SNP genotypes between the hybrid and its parents	3,393	3,847
SNP genotypes specific to the hybrid	4,988	5,534
SNP genotypes consistent with RRIM 600	9,128	7,868
SNP genotypes consistent with PR 107	7,366	8,626
Concordant small InDel genotypes between the hybrid and its parents	21	21
InDel genotypes specific to the hybrid	24	24
InDel genotypes consistent with RRIM 600	30	38
InDel genotypes consistent with PR 107	38	30

**Table 6:** Genotypes of 24 rubber tree varieties corresponding to 10 SNPs.

gene ID	rt_contig_est_22684			rt_contig_est_24190		rt_contig_est_26126			rt_contig_est_959		The heterozygous SNPs percentage	The homozygous SNPs percentage
position	466	546	549	1039	937	1149	1269	1501	1570	885		
ref	T	C	C	C	A	G	G	T	A	G		
alt	C	G	G	T	G	A	A	A	T	A		
BT 3410	0/1	0/1	0/1	0/1	0/1	0/1	0/1	0/1	0/0	0/0	80%	20%
PR 261	0/1	0/1	0/1	0/1	0/1	0/1	0/1	0/1	0/0	0/0	80%	20%
RY 6231	0/1	0/1	0/1	0/1	0/1	0/1	0/1	0/1	0/0	0/0	80%	20%
RY 618	0/1	0/1	0/1	0/1	0/1	0/1	0/1	0/1	0/1	0/0	90%	10%
DL 2138	0/1	0/1	0/1	0/1	0/1	0/1	0/1	0/1	0/0	0/0	80%	20%
DF 318	0/1	0/1	0/1	0/1	0/1	0/1	0/1	0/1	0/0	0/0	80%	20%
DF 99	0/1	0/1	0/1	0/1	0/1	0/1	0/1	1/1	0/0	0/0	70%	30%
BT 911	0/1	0/1	0/1	0/1	0/1	0/1	0/1	0/1	0/1	0/0	90%	10%
DF 95	1/1	0/1	0/1	0/1	0/1	0/1	0/1	0/1	0/1	0/1	90%	10%
DF 7825	1/1	0/0	0/0	0/0	0/0	0/0	0/0	0/0	1/1	0/0	0%	100%
WC 11	0/1	0/1	0/1	0/1	0/1	0/1	0/1	0/1	0/0	0/0	80%	20%
HK 2	0/1	0/1	0/1	0/1	0/1	0/1	0/1	0/1	0/1	0/0	90%	10%
DL 2165	0/1	0/1	0/1	0/1	0/1	0/1	0/1	0/1	0/1	0/0	90%	10%
RRIC 36	0/1	0/1	0/1	0/1	0/1	0/1	0/1	0/1	0/1	0/0	90%	10%
PR 303	0/1	0/1	0/1	0/1	0/1	0/1	0/1	0/1	0/1	0/1	100%	0%
Tjir 1	0/1	0/1	0/1	0/1	0/0	0/0	0/0	0/0	1/1	0/0	40%	60%
BT 155	0/1	0/1	0/1	0/1	0/0	0/0	0/0	0/0	0/0	0/1	50%	50%
BT 235	0/1	0/1	0/1	0/0	1/1	0/0	0/0	0/0	0/0	0/0	30%	70%
HK 6	0/1	0/1	0/1	0/1	0/1	0/1	0/1	0/1	0/0	0/1	90%	10%
PB 86	0/0	0/0	0/0	0/0	1/1	1/1	1/1	1/1	0/0	0/0	0%	100%
RY 7-33-97	0/1	0/1	0/1	0/1	0/1	0/1	0/1	0/1	0/1	0/0	90%	10%
RY 7-20-59	0/1	0/1	0/1	0/1	0/0	0/0	0/0	0/0	0/1	0/1	60%	40%
RRIM 600	0/0	0/0	0/0	0/0	0/1	0/1	0/1	0/1	0/1	0/0	50%	50%
PR 107	0/1	0/1	0/1	1/1	0/0	0/0	0/0	0/0	0/1	0/1	50%	50%
PIC	0.2918	0.2188	0.2188	0.3438	0.448	0.4341	0.4341	0.4862	0.5729	0.375		

BT, RY, DL, DF, HK, and WC represent the abbreviations of Baoting, Reyan, Daling, Dafeng, Haiken and Wenchang rubber trees, and these varieties are bred in China. The genotypes of 0/0, 0/1, and 1/1 represent ref/ref, ref/alt or alt/ref, and alt/alt, respectively.

**Table 7:** Characteristics of genetic variations involved in ET, JA, and rubber biosynthesis/flow pathways between the parents and their hybrids.

Rubber biosynthesis and flow pathway		
The characteristics of genetic variations	Rubber tree hybrids	
	RY 7-33-97	RY 7-20-59
concordant SNP genotypes between the hybrid and its parents	12	14
SNP genotypes specific to the hybrid	3	1
SNP genotypes consistent with RRIM 600	11	4
SNP genotypes consistent with PR 107	7	14
ethylene pathway		
concordant SNP genotypes between the hybrid and its parents	4	4
SNP genotypes specific to the hybrid	20	22
SNP genotypes consistent with RRIM 600	22	20
SNP genotypes consistent with PR 107	1	1
JA pathway		
SNP genotypes specific to the hybrid	1	1
SNP genotypes consistent with RRIM 600	9	11
SNP genotypes consistent with PR 107	8	6
