# Supplementary material for: Gene expression analysis and SNP/InDel discovery to investigate yield heterosis of two rubber tree F1 hybrids
Source: Sci Rep. 2016 Apr 25;6:24984. doi: 10.1038/srep24984 (PMC4842955; doi:10.1038/srep24984)
Supplement: Supplementary Information [file srep24984-s1.pdf]

Supplementary Information

Gene expression analysis and SNP/InDel discovery to investigate yield heterosis of two rubber tree F_1 hybrids

Dejun Li[1,+], Rizhong Zeng[1,+], Yan Li[2,+], Manman Zhao[1,3], Jinquan Chao[1], Yu Li[1], Kai Wang[2], Lihuang Zhu[2,*], Wei-Min Tian[1,*], and Chengzhi Liang[2,*]

[1] Rubber Research Institute, Chinese Academy of Tropical Agricultural Sciences, Danzhou 571737, Hainan, China

[2] State Key Laboratory of Plant Genomics and National Plant Gene Research Centre (Beijing), Institute of Genetics and Developmental Biology, Chinese Academy of Sciences, 5 Datun Road, Chaoyang District, 100101 Beijing, China

[3] College of Horticulture & Forestry Sciences, Huazhong Agricultural University, Wuhan, 430070 China

[+] These authors contributed equally to this work.

*Corresponding author: Email: lhzhu@genetics.ac.cn, wmtian@163.com, cliang@genetics.ac.cn; Tel: 86-10-64801262; Fax: 86-10-64801262

Supplementary table legends

Supplemental Dataset 1 Genes associated with ET and JA responses.

Supplemental Dataset 2 Genes involved in ET and JA pathways.

Supplemental Dataset 3 SNPs and small InDels between RRIM 600 and PR 107.

Supplemental Dataset 4 SNPs and small InDels among four rubber tree varieties.

Supplemental Dataset 5 Validation results of small InDels and SNPs among four rubber tree varieties.

Supplemental Dataset 6 SNPs in genes involved in ET, JA, and latex biosynthesis/flow pathways between the two hybrids and their parents.

Supplemental Dataset 7 Primers designed for validation of SNPs.

Supplemental Dataset 8 Genes involved in latex biosynthesis/flow and their accession numbers in GenBank.

Supplemental Dataset 9 Primers for analyzing gene expression.
